# Cardio-Protective Effects of Multiport Antegrade Cold Blood Cardioplegia Versus Antegrade Cold Blood Cardioplegia in Patients With Left Ventricular Systolic Dysfunction Undergoing Conventional Coronary Artery Bypass Grafting

**DOI:** 10.7759/cureus.10308

**Published:** 2020-09-08

**Authors:** Muhammad Ali, Muhammad Moeen, Iftikhar Paras, Waqas Hamid, Saadat Khan, Muhammad Hamid Chaudhary

**Affiliations:** 1 Cardiac Surgery, Chaudhry Pervaiz Elahi Institute of Cardiology, Multan, PAK; 2 Cardiac Surgery, Sheikh Zayed Medical College/Hospital, Rahim Yar Khan, PAK; 3 Echocardiography, Tabba Heart Institute, Karachi, PAK

**Keywords:** left ventricular systolic dysfunction, coronary artery bypass grafting, multiport antegrade cold blood cardioplegia

## Abstract

Introduction

The aim of this study was to compare the in-hospital outcomes of multiport antegrade cold blood cardioplegia through vein grafts versus conventional antegrade cold blood cardioplegia in patients with left ventricle systolic dysfunction who underwent coronary artery bypass grafting (CABG).

Methods

This prospective, randomized clinical study was comprised of patients undergoing on-pump CABG at the Ch. Pervaiz Elahi Institute of Cardiology in Multan, Pakistan from November 18, 2018 to December 17, 2019. Patients with multivessel coronary artery disease and left ventricular systolic dysfunction (ejection fraction < 50%) were included. In Group A (N = 73), multiport antegrade cold blood vein graft cardioplegia was given after every distal anastomosis completed for myocardial preservation. In Group B (N = 73), conventional antegrade cold blood cardioplegia was given for myocardial preservation.

Results

Spontaneous rhythm (without defibrillation applied) after cross-clamp removal was higher in Group A than in Group B (93.3% vs. 85.2%, p < 0.05). Duration of support, ventilation time, and hospital stay were also significantly lower in Group A than in Group B with p = 0.00001, p = 0.03, and p = 0.002, respectively. Intra-aortic balloon pump insertion (4.1% vs. 23.0%, p = 0.02) and operative mortality (0.5% vs. 4.0%, p = 0.35) were also lower in Group A than in Group B. Postoperative left ventricular ejection fraction (LVEF) increased more in Group A than in Group B, and the postoperative LVEF mean value was 44.68% in Group A versus 41.26% in Group B (p = 0.02).

Conclusion

Multiport vein graft blood cardioplegia provides superior myocardial protection in patients with left ventricular systolic dysfunction who underwent CABG. It is also easy to administer, so this technique can be adopted as a routine method for myocardial protection in patients with left ventricular dysfunction planned for on-pump CABG.

## Introduction

Performing coronary artery bypass grafting (CABG) on cardiopulmonary bypass (CPB) is considered the gold standard [1]. In the United States, more than 80% of CABG procedures are performed using CPB [2]. Antegrade cardioplegia through aortic root is the most well-known method of myocardial protection. Antegrade cardioplegia provides quicker diastolic arrest and preserves myocardial function. Most cardiac centers prefer antegrade cardioplegia due to its safety, simplicity, and superiority to other methods [1]. In severely diseased coronary arteries, antegrade cardioplegia does not perfuse beyond the occluded artery, which leads to suboptimal protection to the myocardium and low ejection fraction (EF) postoperatively. Optimal myocardial protection in poor left ventricular (LV) function patients can be achieved by adequate distribution of cardioplegia to all areas of the heart and minimization of ischemia time [3,4]. Maldistribution of cardioplegia can be avoided by injection through antegrade and retrograde. Antegrade cardioplegia infused in the aortic root and retrograde through the coronary sinus provides marginal protection in severely occluded arteries and low LV function patients [5,6]. Retrograde cardioplegia has limitations that do not protect the right ventricle of the heart and introduce the risk of coronary sinus damage [6]. To address these challenges, in a severely occluded coronary artery, it is recommended to perfuse the cold blood cardioplegia in the aortic root as well as in the vein graft through multiport cardioplegia cannula, also known as the ‘multiport antegrade cold blood cardioplegia’ technique, which is a harmless and simple technique [7].

This study was designed to compare in-hospital outcomes of multiport antegrade cold blood cardioplegia through vein grafts versus conventional antegrade cold blood cardioplegia in patients with LV systolic dysfunction who underwent CABG.

## Materials and methods

This prospective, randomized clinical study was composed of patients undergoing CABG on CPB at the Ch. Pervaiz Elahi Institute of Cardiology in Multan, Pakistan from November 18, 2018 to December 17, 2019. Patients’ informed consent signatures were collected and approved by the research evaluation unit of the College of Physicians and Surgeons of Pakistan. The study population included all patients who were admitted for CABG in the Department of Cardiac Surgery of Ch. Pervaiz Elahi Institute of Cardiology.

This study included men and women of all ages with multivessel coronary artery disease (e.g., double-vessel or triple-vessel coronary artery disease) and LV systolic dysfunction (i.e., patients with EF < 50% were included). Exclusion criteria included patients with single-vessel coronary artery disease (CAD), normal biventricular systolic function (i.e., with EF > 50%), or clinically significant valvular disease (i.e., more than mild valvular disease). Patients with less than 1 mm target vessels or diffuse coronaries, reoperations, and a history of cerebrovascular disease were also excluded.

Randomization was applied to categorize the patients into two equal groups (Group A and Group B). Randomization was done using lottery method. We made 146 folded papers containing name of intervention (73 folded papers for each intervention) and put them in a jar. Every time before taking the patient to operating room, the staff nurse on duty was asked to pick one folded paper. The patients were randomized on the basis of folded paper chosen by the staff nurse. In Group A, multiport antegrade cold blood vein graft cardioplegia was given after every distal anastomosis was completed for myocardial preservation. In Group B, conventional antegrade cold blood cardioplegia was given for myocardial preservation.

All CABG procedures were done by two consultant surgeons at the institute. All patients followed regular preoperative medications on the day of surgery, including bromazepam 3 mg orally. For induction of anesthesia, morphine, midazolam, and propofol were administered, and doses were adjusted depending on patients’ responses. Atracurium bromide (1 mg/kg) was injected before intubation.

In all patients, the chest was exposed through a median sternotomy. After harvesting of the left internal mammary artery and radial artery or saphenous vein conduit, 4 mg/kg of heparin was given before cannulation to maintain above 480 seconds of activated clotting time. Standard conventional CPB was established with ascending aortic cannulation, two-staged venous cannula, and regular vent-port cardioplegia cannula insertion. Hypothermia applied to 30°C to 32°C. After aortic cross-clamp, 4°C to 8°C cold blood cardioplegia (blood:crystalloid cardioplegia, 4:1) was infused through the cardioplegia delivery system into the coronary arteries to arrest the heart. An initial dose of 20 mL/kg cardioplegia was given to achieve a complete arrest.

In Group A, after distal anastomosis of each vein graft, a free branch of a multiple perfusion set was attached to the proximal end of the vein graft for infusing the cardioplegia. A 10-mL/kg maintenance dose cardioplegia was delivered in a multiple perfusion set along with cardioplegia cannula. Until anastomosis of all distal vein grafts was completed, multiport cold blood cardioplegia was delivered simultaneously with antegrade cardioplegia. In Group B, a maintenance dose of 10 mL/kg of cold blood cardioplegia was repeated after every graft or every 20 minutes (whichever was first or applied) through cardioplegia cannula. After cross-clamp removal, proximal anastomosis was performed after hemodynamic stability was achieved. Smooth wean off was done with or without ionotropic support. All patients were followed until one month after surgery to determine the operative outcomes.

Statistical analysis was done using IBM SPSS Statistics for Windows, Version 23.0. (Armonk, NY, IBM Corp.). Quantitative data were analyzed using means ± standard deviation, and qualitative data were analyzed using a chi-square test. The group comparison was conducted by an independent sample t-test, and the statistical significance among the group was represented using a p value ≤ 0.05.

## Results

There was no significant difference among the groups regarding age, gender, diabetes, hyperlipidemia, preoperative left ventricular ejection fraction (LVEF), and preoperative creatinine kinase myocardial band (CKMB) levels (p > 0.05). There was no difference in the number of diseased vessels, and patients in both groups had left ventricle systolic dysfunction preoperatively (Table 1).

**Table 1:** Baseline demographic and operative characteristics Abbreviations: TVD, triple-vessel disease; DVD, double-vessel disease; LVEF, left ventricle ejection fraction; CKMB, creatinine kinase myocardial band.

Characteristics	Group A Study Group (N = 73)	Group B Control Group (N = 73)	p Values
Age (years)	53.20 ± 8.2	54.20 ± 6.32	0.41
Men/women	62 (84.9%)/11 (15.1%)	64 (87.7%)/9 (12.3%)	0.63
Family history (%)	10 (13.7%)	11 (15.1%)	0.81
Diabetes mellitus (%)	40 (54.8%)	32 (43.8%)	0.18
Smoking (%)	42 (57.5%)	50 (68.5%)	0.17
Hypertension	47 (64.4%)	45 (61.6%)	0.73
Hyperlipidemia (%)	11 (15.1%)	9 (12.3%)	0.63
Number of diseased vessels (%) TVD, DVD	49 (67.1%), 24 (32.9%)	46 (63.0%), 27 (37.0%)	0.60
Preoperative LVEF (%)	38.42 ± 6.39	39.86 ± 5.58	0.14
Preoperative CKMB level	20.80 ± 10.57	20.70 ± 10.48	0.95

Intra-operative data and postoperative results are shown in Table 2. There was a highly significant difference noted between the groups regarding bypass time (p = 0.001). In Group A (study group), the CPB time was less than that in Group B (control group).

**Table 2:** Comparison of intra-operative and postoperative outcomes Abbreviations: SD, standard deviation; LVEF, left ventricle ejection fraction; CKMB, creatinine kinase myocardial band; IABP, intra-aortic balloon pump; IU, international unit; sig, significant difference.

Characteristics	Group A Study Group (N = 73)	Group B Control Group (N = 73)	p Values
Bypass time (minutes)	118.90 ± 29.20	128 ± 34.23	0.001 (sig)
x-clamp time (minutes)	62.22 ± 14.46	69 ± 16.40	0.08
No. of grafts (mean ± SD)	3.36 ± 0.50	3.31 ± 0.55	0.06
Postoperative CKMB level (IU)	68.54 ± 42.68	98.62 ± 74.63	0.002 (sig)
Duration of support (hours)	6.11 ± 9.17	10.10 ± 15.62	0.0001 (sig)
Ventilation time (hours)	5.60 ± 7.04	09 ± 25.57	0.03 (sig)
Hospital stay time (days)	6.69 ± 1.22	6.62 ± 2.57	0.002 (sig)
IABP (%)	3 (4.1)	13 (23.0)	0.02 (sig)
Spontaneous rhythm (%)	93.3	85.2	0.004 (sig)
Operative mortality (%)	0.5	4.0	0.35
Postoperative LVEF (%)	44.68	41.26	0.02 (sig)

Spontaneous rhythm (without defibrillation applied) after cross-clamp removal was higher in Group A than in Group B (93.3% vs. 85.2%, p < 0.05). Duration of support, ventilation time, and hospital stay were also significantly lower in Group A than in Group B with p = 0.00001, p = 0.03, and p = 0.002, respectively. Operative mortality (0.5% vs. 4.0%, p = 0.35) and intra-aortic balloon pump (IABP) insertion (4.1% vs. 23.0%, p = 0.02) were also lower in Group A than in Group B. Postoperative LVEF increased more in Group A than in Group B, with a postoperative LVEF mean value of 44.68% in Group A versus 41.26% in Group B (p = 0.02). Postoperative peak CKMB level was statistically considerably higher (p = 0.002) in Group B compared to that in Group A (Table 2). 

## Discussion

With the progression of medical technology, percutaneous intervention methodology for the treatment for CAD is considered conventional therapy; however, in extreme multivessel coronary disease, patients were referred for CABG. The primary principle of CABG is to provide adequate blood supply to the ischemic areas through revascularization. Hence, several improvements in cardioplegia delivery techniques have been made to improve myocardial preservation [8,9]. As there is a high probability of postoperative mortality in CABG due to myocardial dysfunction [10,11], myocardial protection during CPB can be achieved by providing adequate distribution of a cardioplegia solution throughout the myocardium [12]. Distribution of cardioplegia beyond the occluded coronary vessels effectively protects the myocardium [13].

To date, the combination of antegrade and retrograde cardioplegia infusion is considered the gold standard in myocardial protection [14]. Conventional cold blood antegrade root cardioplegia is considered an effective method due to its safety and simplicity.

This study method was nearly identical to the method suggested by Goldman et al., which stated that careful graft perfusion provides appropriate cooling of the most ischemic zones to protect myocardium by reperfusion of cold blood cardioplegia. The researchers concluded that this method is suitable for patients who present with acute ischemia, failed angioplasty, left main occlusion, and LV systolic dysfunction [15].

Until now, not many studies have been done on cardioplegia through vein grafts to protect the myocardium. There are conflicting results found in different studies about the present method. Onem et al. showed that there was no advantage of perfusing the antegrade cold blood cardioplegia through vein grafts in low-risk patients [13]. Other published studies demonstrated that there was no benefit to myocardium preservation when using simultaneous antegrade root and vein graft cardioplegia together. Both studies had a small sample size and excluded poor LVEF patients [16,17].

Our study results were almost the same as an earlier randomized controlled study conducted by Sher-I-Murtaza et al., who reported lower cardiac troponin I levels when patients were given passive multiport cold blood cardioplegia, which indicates it is superior to conventional cardioplegia [7]. The lower bypass time, lower incidence of arrhythmia after x-clamp removal, lower IABP insertion count, lower postoperative CKMB*isoenzyme level (IU) release, and the increases in postoperative LVEF were all good signs of myocardial protection in the study group. Our study found that antegrade cold blood graft cardioplegia provided superior myocardial preservation in LV systolic dysfunction due to proper cardioplegia distribution in even proximal occluded artery.

## Conclusions

Multiport vein graft infusion of blood cardioplegia provides superior myocardial protection in CABG patients having preoperative LV systolic dysfunction. Due to its ease of administration, this technique can be adopted as a routine method for myocardial protection in patients with LV dysfunction planned for on-pump CABG.
